# Addressing mood and fatigue in return-to-work programmes after stroke: a systematic review

**DOI:** 10.3389/fneur.2023.1145705

**Published:** 2023-08-22

**Authors:** Nicole Yun Ching Chen, YanHong Dong, Zaylea Zhong Jie Kua

**Affiliations:** ^1^Changi General Hospital, Singapore, Singapore; ^2^Department of Medicine, Yong Loo Lin School of Medicine, National University of Singapore, Singapore, Singapore; ^3^Alice Lee Centre for Nursing Studies, Yong Loo Lin School of Medicine, National University of Singapore, Singapore, Singapore

**Keywords:** systematic review, stroke rehabilitation, return-to-work, mood, fatigue

## Abstract

**Introduction:**

Return-to-work is a key rehabilitation goal for many working aged stroke survivors, promoting an overall improvement of quality of life, social integration, and emotional wellbeing. Conversely, the failure to return-to-work contributes to a loss of identity, lowered self-esteem, social isolation, poorer quality of life and health outcomes. Return-to-work programmes have largely focused on physical and vocational rehabilitation, while neglecting to include mood and fatigue management. This is despite the knowledge that stroke results in changes in physical, cognitive, and emotional functioning, which all impact one’s ability to return to work. The purpose of this systematic review is to conduct a comprehensive and up-to-date search of randomised controlled trials (RCTs) of return-to-work programmes after stroke. The focus is especially on examining components of mood and fatigue if they were included, and to also report on the screening tools used to measure mood and fatigue.

**Method:**

Searches were performed using 7 electronic databases for RCTs published in English from inception to 4 January 2023. A narrative synthesis of intervention design and outcomes was provided.

**Results:**

The search yielded 5 RCTs that satisfied the selection criteria (*n* = 626). Three studies included components of mood and fatigue management in the intervention, of which 2 studies found a higher percentage of subjects in the intervention group returning to work compared to those in the control group. The remaining 2 studies which did not include components of mood and fatigue management did not find any significant differences in return-to-work rates between the intervention and control groups. Screening tools to assess mood or fatigue were included in 3 studies.

**Conclusion:**

Overall, the findings demonstrated that mood and fatigue are poorly addressed in rehabilitation programmes aimed at improving return-to-work after stroke, despite being a significant predictor of return-to-work. There is limited and inconsistent use of mood and fatigue screening tools. The findings were generally able to provide guidance and recommendations in the development of a stroke rehabilitation programme for return-to-work, highlighting the need to include components addressing and measuring psychological support and fatigue management.

## 1. Introduction

Stroke is defined as “rapidly developing clinical signs of focal (or global) disturbance of cerebral function, lasting more than 24 h or leading to death, with no apparent cause other than that of vascular origin” (1). It is the second-leading cause of death and third-leading cause of death and disability combined (as measured by disability-adjusted life-years) with rising trends and overall global public health burden (2).

Approximately 10% of all strokes occur in individuals aged below 50 years (3, 4). The hospitalisation rates of acute ischemic stroke among those aged 25 to 44 have increased considerably from 2000 to 2010 by 44% in the United States, despite an overall decline (5). Stroke incidence has been increasing in young adults in developing countries due to: improvements in stroke detection, increase in vascular risk factors (e.g., alcohol consumption, smoking, hypercholesterolemia, obesity) in young adults, and potentially environmental factors (e.g., air pollution) (4).

As younger adults are responsible for supporting family and generating income, a key rehabilitation goal is in their ability to return to work. Stroke survivors who return to paid work have shown improved psychosocial outcomes (6), subjective wellbeing, and life satisfaction (7, 8). Conversely, the failure to return to work following stroke contributes not only to a loss of identity, lowered self-esteem, quality of life and poorer health outcomes for younger stroke survivors, but also increases socioeconomic burdens arising from the loss of work productivity (9, 10).

Return-to-work programmes have largely focused on physical and vocational rehabilitation, while neglecting to include mood (e.g., depression, anxiety, stress) and fatigue management. This is despite the knowledge that stroke results in changes in physical, cognitive, and emotional functioning, which all impact one’s ability to return to work. A review of the literature on return-to-work after stroke found that the rehabilitation process involves multiple predictors for its success, including physical factors (stroke severity, functional disability), social factors (ethnicity, income, gender, occupation) and cognitive/emotional factors (psychiatric disorders, fatigue, cognitive functioning) (11). A study found that psychiatric morbidity was a significant determinant of return to paid work after stroke, hence authors recommended appropriate management of the emotional consequences of stroke, suggesting that this would optimise recovery and enable successful return-to-work in working aged stroke survivors (12). Fatigue, which is closely related to mood, has also been found to be a significant barrier to return-to-work after stroke (13, 14).

Research has shown the predictive effect of mood and fatigue on return-to-work after stroke, yet there is no published research examining mood and fatigue components in return-to-work intervention programmes after stroke. Prior systematic reviews on return-to-work programmes after stroke have largely found studies of poor quality, heterogeneity in methodology and limitations of inadequate search, highlighting the need to examine high quality randomised controlled trials (RCTs) (8, 15). Hence, this study sought to examine solely RCTs to address this. The aim of this systematic review is to conduct a comprehensive and up-to-date search of RCTs of return-to-work programmes after stroke. The focus is especially on examining components of mood and fatigue, if they were included.

## 2. Methods

This review is registered with PROSPERO with registration number CRD42023388567.^1^ It is reported in accordance with the Preferred Reporting Items for Systematic Reviews and Meta-Analyses statement (16).

### 2.1. Eligibility criteria

All studies yielded in response to the search terms were identified against the inclusion and exclusion criteria. The inclusion criteria were RCTs published from inception to 4 January 2023, studies published in English, include a population of stroke survivors (16–85 years old), with one of the primary outcomes of rehabilitation being return-to-work (including paid work, unpaid work, volunteering, housework). Eligible studies included interventions of an RCT design, of any type and duration against an active or passive control group. Examples include cognitive training/rehabilitation, digital interventions (computerised, application-based), and vocational rehabilitation.

Studies not eligible included: participants of other diagnostic groups or with mixed etiologies (e.g., traumatic brain injury/stroke mix) and interventions are not sufficiently detailed (e.g., description of intervention, specific components, dosage and frequency of sessions). Qualitative studies, previous systematic reviews or meta-analyses were excluded.

### 2.2. Information sources

Searches were conducted using electronic databases (Medline, PubMed, Embase, PsycInfo, Scopus, Web of Science, Cochrane Central Register of Controlled Trials) and manual search. The search terms used to identify relevant articles included “stroke,” “cerebrovascular accident,” “cerebral infarction” or “brain attack” or “apoplexy,” “return to work,” “employment,” or “job,” “rehabilitation,” “training,” “programme,” “intervention” or “protocol,” and “randomized controlled trial,” “controlled clinical trial,” “randomized,” “trial,” “groups” or “double blind.” We combined the search terms using Boolean operators “AND” and “OR.” A manual search of the reference lists for relevant studies was also undertaken to identify studies that were overlooked in the electronic search.

### 2.3. Search strategy

Full search strategies for all the databases are included in Supplementary Appendix A. The search was separately conducted and compared by 2 authors (N.Y.C.C and Z.Z.J.K).

### 2.4. Selection process

After the search and removal of duplicates, studies were screened using the eligibility criteria specified above. Titles that were irrelevant were eliminated. Abstracts for the remaining studies were reviewed based on the criteria, with full text reviewed if the abstract did not provide sufficient information. The final selection of studies was independently determined and agreed on by 2 authors (N.Y.C.C and Z.Z.J.K). Discrepant views were discussed and decided with the third reviewer (Y.D).

### 2.5. Data collection process

Relevant data were extracted and recorded in tables to illustrate the characteristics of the included studies (Table 1). Data extracted included characteristics of participants (age, gender, time since onset of stroke), mood and fatigue components in intervention, mood/fatigue measures, and outcomes of intervention.

**Table 1:** Characteristics of reviewed studies.

Study	*n*	Mean Age (SD)	Mean time since most recent stroke before intervention	Mood and fatigue component in intervention	Measure of mood/fatigue	Outcomes of intervention
Cain et al., 2022 *Information obtained from Bernhardt et al.*, (2015)*	*I* = 200 *C* = 176	56	*I* = 18.5 h *C* = 22.4 h	Not included	Irritability Depression Anxiety Scale	Univariate analysis showed no significant differences in the odds of returning to work between the intervention and control group (OR = 1.33, 95% CI [0.88–2.01], *p* = 0.18). Odds of returning to work were increased with less depressive traits at 3 months (OR = 0.87, 95% CI [0.80–0.93], *p* < 0.001). Among the combined cohort, multivariate analysis found age (OR = 0.94, 95% CI [0.91–0.98], *p* < 0.001), stroke severity (OR = 0.92, 95% CI [0.86–0.99], *p* < 0.02), 3-month disability, and full-time work before stroke (OR = 2.3, 95% CI [1.24–4.40], *p* < 0.009) to be significantly associated with return-to-work.
Ghoshchi et al., 2020	*I* = 23 *C* = 25	51.0 (11.8) 52.5 (10.5)	*I* = 27.0 months *C* = 21.7 months	Not included	Not included	No significant differences were found between the intervention and control group in terms of the number of subjects who returned to work (*p* < 0.406). Regression analyses found that the Modified Barthel Index score at follow-up significantly influenced return-to-work (OR = 7.5, 95% CI [2.04–27.59], *p* < 0.002).
Mead et al., 2022	*I* = 39 *C* = 37	67.3 (12.5) 66.1 (4.3)	*I* = 10.3 months *C* = 10.2 months	Provision of psychoeducation on fatigue, overcoming fears and increasing physical activity, cognitive restructuring, and addressing unhelpful thoughts related to fatigue and low mood.	Fatigue Assessment Scale, Generalised Anxiety Disorder-7	There was no differences in return-to-work between groups. There was no statistically significant differences between groups in six-month Fatigue Assessment Scale, Patient Health Questionnaire-9, and Generalised Anxiety Disorder-7. The small negative mean differences indicated that the intervention was slightly better than the control.
Ntsiea et al., 2014	*I* = 40 *C* = 40	45.0 (8.5) 44.0 (8.9)	I & C < 8 weeks (in order to start intervention before the end of 6-week sick leave period)	Provision and discussion of emotional support, coping techniques, and fatigue management. A psychologist or social worker was involved if necessary.	Not included	At 6 months follow-up, 60% of subjects in the intervention group had returned to work compared to 20% in the control group, *p* < 0.001. The likelihood of returning to work were higher for subjects with greater functional independence in activities of daily living (OR = 1.7, 95% CI [1.10–2.60], *p* = 0.02) and higher cognitive scores (OR = 1.3, 95% CI [1.10–1.60], *p* = 0.02) at 6 months follow-up. Fatigue was one of the reasons for subjects not returning to work.
Radford et al., 2020 *Information obtained from Radford et al.*, (2014)*	*I* = 23 *C* = 23	58.3 (12.7) 53.8 (12.6)	Not stated	Psychological support was provided to assist with adjustment post-stroke, preparing for work, and throughout the return-to-work process. Participants were provided resources if they needed more psychological, social support, or medical assistance.	Hospital Anxiety and Depression Scale	Descriptive statistics showed a higher percentage of subjects who received the intervention (37.5%) reporting full-time work at 12 months follow-up, compared to controls (11.8%). Workplace accommodations were more common among subjects from the intervention group compared to the control group. More subjects from the intervention group reported satisfaction with work at both 6 and 12 months post-randomisation compared to the control group.

C, control group; I, intervention group.

### 2.6. Assessment of risk of bias in included studies

The selected studies were assessed for risk of bias using the Cochrane Collaboration’s tool for assessing risk of bias in randomised trials revised version (RoB 2) (17). The effect of interest was the effect of assignment to the interventions at baseline, estimated by the intention-to-treat analysis. Outcome domain of interest is participants’ return-to-work. Domains in RoB 2 included the following biases: the randomisation process, deviations from intended interventions or missing outcome data, outcome measurement, and selection of the reported results. The risk of bias assessment was performed independently by N.Y.C.C and Z.Z.J.K. Disagreement was resolved by discussion and reaching consensus. Signalling questions were used to determine the levels of bias (high risk, some concerns and low risk) assigned to each domain.

## 3. Results

### 3.1. Study selection

**Figure 1:**
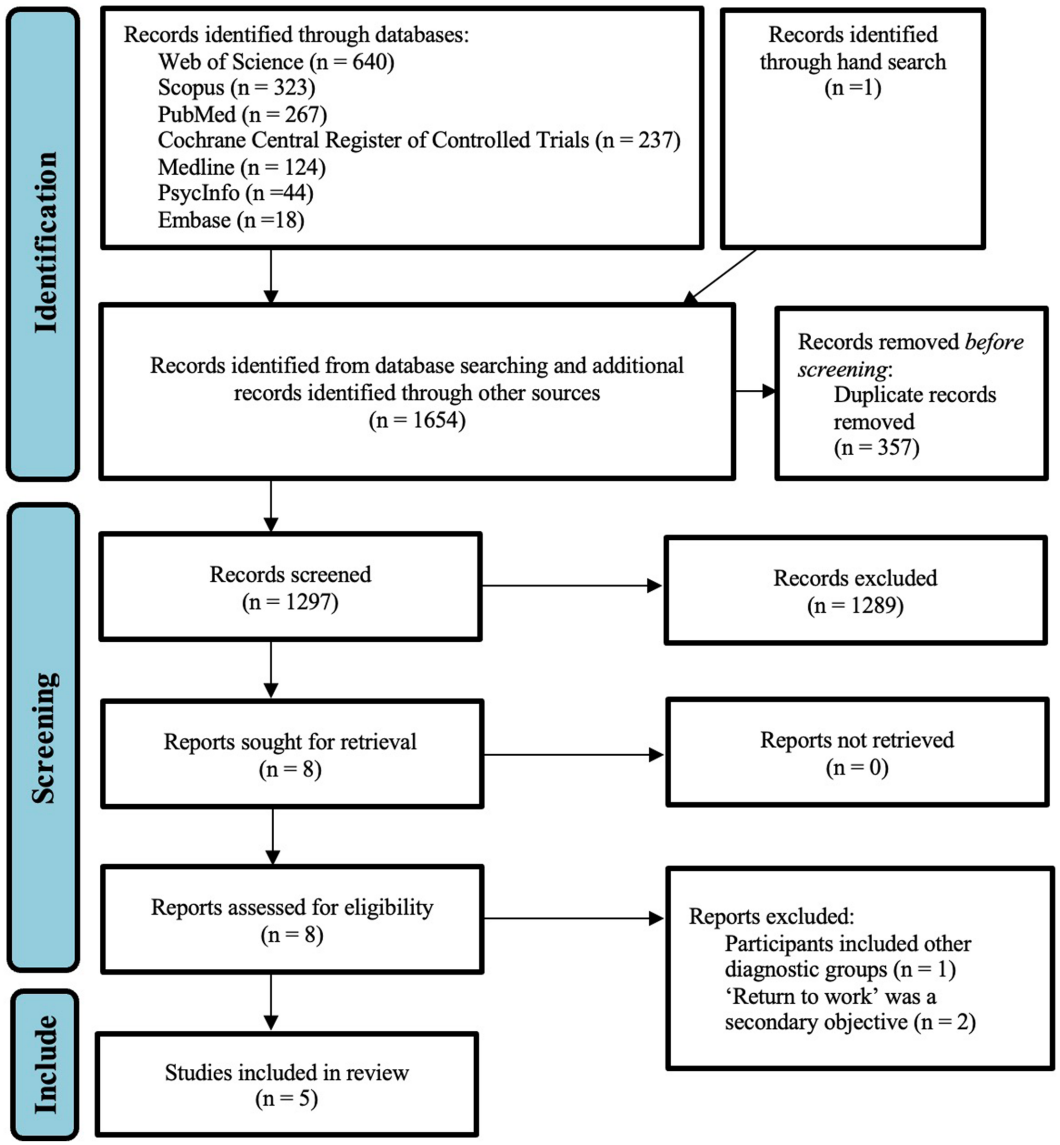
PRISMA flow diagram (15).

The search yielded a total of 1,654 articles. After removing duplicates, 1,297 articles were screened using the titles and abstracts, of which 1,289 articles were discarded. The full text of the remaining 8 articles was reviewed and 3 articles were excluded, with reasons such as other diagnostic group besides stroke (i.e., traumatic brain injury) and return-to-work being a secondary objective. Five final articles were included in the final review (see Figure 1).

### 3.2. Study characteristics

Table 1 provides the characteristics of the included studies, which were all randomised controlled trials on stroke rehabilitation. Four of the five studies were recently published between 2020 and 2022 (18–21). The total number of subjects included in these 5 studies is 626, with sample size ranging from 46 to 376. Only 1 study had a sample size larger than 100 (18), while the other 4 studies had fewer than 100 subjects (19–22). The mean age ranged between 44 and 66.7. Cain et al. (18) and Ntsiea et al. (22) defined “work” as paid formal employment, while Radford et al. (21) included paid work, unpaid (voluntary) work and full-time education. Ghoshchi et al. (20) and Mead et al. (19) did not provide definitions of “work.” The studies generally included information on the diagnosis of stroke, age, gender ratio and employment at the time of stroke. Most studies provided additional information such as follow-up period and time since onset of stroke.

### 3.3. Risk of bias in included studies

Two papers were sub-studies of large randomised controlled trials, where their primary results and methodology were published in another paper (18, 21). The original protocols were retrieved to assess for risk of bias.

Table 2 depicts the risk of bias assessment. Three studies were judged to be at low risk of bias for all domains (18, 19, 22). The other two studies were assessed to raise some concerns. In Ghoshchi et al. (20), no information was provided regarding allocation concealment, participants’ awareness of their assigned intervention and if there were any deviations from the intended intervention. However, given that all participants who were randomised were included in the analyses, the overall risk of bias was judged to raise some concerns. In Radford et al. (21), outcome data was missing for 13% of participants, of which there were more control participants with missing data than intervention participants. Grant et al. (25) acknowledged that there may be bias of results towards the intervention group due to more knowledge known about their vocational status than the control group, raising high risk of bias in the domain of missing outcome data. However, as this was a feasibility RCT which aimed to evaluate the parameters (e.g., assessing willingness of participants to be randomised, measuring acceptability of intervention) needed to deliver the stroke-specific vocational rehabilitation, the study’s overall risk of bias was maintained at having some concerns.

**Table 2:** Risk of bias assessment.

Studies	Randomisation process	Deviations from the intended interventions	Missing outcome data	Measurement of the outcome	Selection of the reported result	Overall risk-of-bias judgement
Cain et al. (2022)	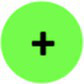 #	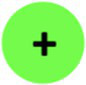 #	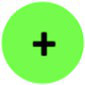 #	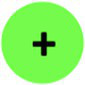 #	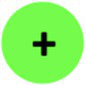	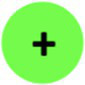
Ghoshchi et al. (2020)	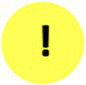	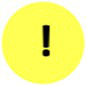	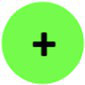	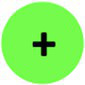	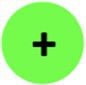	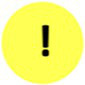
Mead et al. (2022)	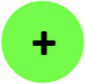	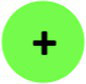	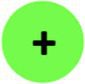	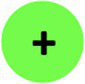	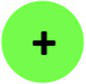	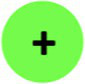
Ntsiea et al. (2015)	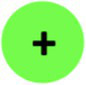	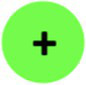	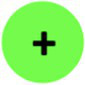	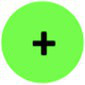	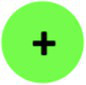	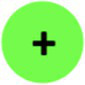
Radford et al. (2020)	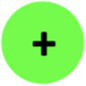 ^	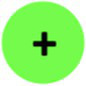 ^	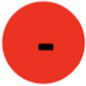 ^	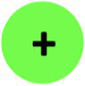 ^	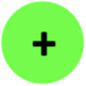	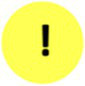

Key: 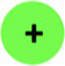 Low risk of bias; 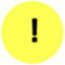 Some concerns of bias; 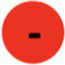 High risk of bias.

For papers which were sub-studies, the original papers were retrieved and reviewed to evaluate the risk of bias.

# Information was obtained from the original study protocol paper (23).

^ Information was obtained from the original study protocol papers (24, 25).

### 3.4. Intervention features

Descriptions of the return-to-work interventions were obtained. In two studies (18, 21), information was obtained from the original papers which included details of the intervention protocol (23–25).

In the largest study (*n* = 376), Cain et al. (18) aimed to describe characteristic of younger working-aged stroke individuals and identify the factors associated with return-to-work at 12 months post-stroke, by comparing early mobility-based rehabilitation to usual care. They included three main components in the intervention: (a) beginning within 24 h of stroke onset, (b) focus on out-of-bed (i.e., sitting, standing, walking) activity, and (c) at least three additional out-of-bed sessions compared to usual care. Trained physiotherapy and nursing staff assisted subjects to continue out-of-bed activity at a frequency according to a detailed intervention protocol, with the frequency adjusted as per the individual’s recovery rate. The intervention duration was 14 days or until discharge from stroke-unit care, depending on which was sooner. The control group received usual care, which were at the discretion of individual sites.

Ghoshchi et al. (20) aimed to assess return to work and quality of life after stroke, utilising technological treatment in their intervention. Both the intervention group (termed Technological Rehabilitation group; TG) and control group (termed Control Conventional Rehabilitation Group; CG) performed 2 sessions of neuromotor rehabilitation per day and 1 session of speech therapy, respiratory or phoniatric rehabilitation. In the TG, 400 min of neuromotor rehabilitation sessions were performed using technological devices (SonicHand or Riablo TM) following specific rehabilitation protocol. SonicHand was administered to subjects with hand deficits requiring rehabilitation in their upper limb fine motor functions and hand dexterity, while Riablo (a videogame-based therapy using wearable sensors to provide biofeedback) was administered to subjects with impaired postural balance and limb gross motor functions. Both devices provided technological biofeedback related to the subjects’ movements. The CG performed rehabilitation sessions according to conventional therapy focusing on hand, balance, or posture, targeting the resumption of independence in activities of daily living. The sessions were conducted in the day hospital, at a frequency of 3 days per week for 1 month, with each session lasting 40 min.

Mead et al. (19) focused on addressing post-stroke fatigue. The intervention sessions included: introduction and psychoeducation on fatigue, goal setting and activity planning, progress assessment and goal modification, cognitive restructuring, dealing with setbacks and barriers, and making future plans. The focus was on encouraging participants to overcome fears of physical activity, increase physical activity using diary monitoring and activity scheduling, achieving balance between activities and rest, and addressing unhelpful thoughts related to fatigue and low mood. The intervention took place over a period of 12 weeks, which included 6 phone calls of an hour each, followed by a booster call 2–4 weeks later. The control group received a leaflet from the national stroke association about post-stroke fatigue.

Ntsiea et al. (22) conducted a workplace intervention programme. The intervention was tailored to individuals’ functional ability and workplace challenges. The intervention started with a work skill assessment for formulating individual treatment plans. Thereafter, sessions took place at the workplace, which included: (a) separate interviews with the subject and employer to identify perceived barriers and motivators of return-to-work; (b) working on identified barriers and discussing a plan for reasonable workplace accommodations (including vocational counselling, coaching, emotional support, workplace adaptation, coping techniques, fatigue management); and (c) monitoring progress of the intervention programme and making adjustments as required. The intervention lasted 6 weeks, with sessions taking place once a week for 1 hour per session, except for work skill assessment sessions which took a minimum of 4 hours. The control group received usual care which included general activities provided by physiotherapists and occupational therapists to improve impairments and limitations to prepare for return home.

Radford et al. (21) conducted an early stroke specific vocational rehabilitation (ESSVR) intervention. Individuals received a mean of 10 sessions, with sessions lasting approximately an hour. ESSVR included assessment of the individual, job analysis, provision of information, education of cognitive and executive functioning skills, advice and psychological support, goal setting, workplace assessment, liaison (with family members, employer, other professionals and services). Psychological support was provided to participants, family members and employers to assist with adjustment following the stroke, work preparation and throughout the return to work process. This involved asking how participants felt during sessions, listening to their concerns and providing encouragement and positive reinforcement as they tried to regain skills and confidence. Work preparation was individualised, including discussion of work options, simulations and interventions (e.g., fatigue management). The control group received usual stroke rehabilitation provided by primary and secondary care, community, and social services, which included rehabilitation for activities of daily living.

### 3.5. Outcomes

Across most studies, the participants were employed at the time of their stroke (18, 20–22). Work status at the respective follow-up time points was reported in all 5 studies. Table 1 summarises the outcomes of the intervention.

Three studies included components of mood and fatigue management in the intervention (19, 21, 22), of which 2 studies found a higher percentage of subjects in the intervention group returning to work compared to those in the control group. In Ntsiea et al. (22), 60% of subjects in the intervention group had returned to work compared to 20% in the control group at 6 months follow-up, which was statistically significant (*p* < 0.001). Furthermore, subjects in the intervention group had 5.2 times higher odds of returning to work at 6 months follow-up than those in the control group (95% CI [1.80–15.0], *p* = 0.002) (22). In addition, fatigue was also found to be one of the main reasons for subjects not returning to work (22). In Radford et al. (21), descriptive statistics showed a higher percentage of subjects in the intervention group (37.5%) reporting full-time work at 12 months follow-up, compared to subjects in the control group (11.8%). Out of those who returned to work by 3 months post-stroke and were able to sustain this until 12 months (*n* = 12), 8 subjects were from the intervention group, compared to 4 subjects from the control group.

The remaining 2 studies which did not include components of mood and fatigue management did not find any significant differences in return-to-work rates between the intervention and control groups (18, 20).

Mood measures were only included in 3 studies (18, 19, 21), namely the Irritability Depression Anxiety Scale, Hospital Anxiety and Depression Scale, Patient Health Questionnaire-9, and Generalised Anxiety Disorder-7. Fatigue measures, namely the Fatigue Assessment Scale, was only included in 1 study (19). Although Ntsiea and colleague’s workplace intervention programme featured elements of mood and fatigue management, it did not include any mood or fatigue measures for screening or measurement of outcome (22).

## 4. Discussion

Return-to-work is an important outcome and rehabilitation goal for many working aged stroke survivors, promoting an overall improvement of quality of life, wellbeing and life satisfaction. Stroke survivors should be well-supported in their reintegration into working life. It is evident that alongside the neurological and physical effects of a stroke, survivors also experience emotional and cognitive changes. These elements have to be addressed in a comprehensive return-to-work rehabilitation programme.

Return-to-work programmes have largely focused on physical and vocational rehabilitation, while neglecting to include mood and fatigue management. Research has shown the predictive effect of mood and fatigue on return-to-work after stroke (26–31), yet there is no published research looking at mood and fatigue components in return-to-work intervention programmes after stroke. This systematic review comprised of a comprehensive and up-to-date search of return-to-work programmes after stroke, specifically examining components of mood and fatigue management. This review concentrated on randomised controlled trials, addressing limitations of previous systematic reviews (8, 15). The included studies were also relatively recent, published between 2015 and 2022.

Depression affects a third of stroke survivors up to 15 years post-stroke, which can continue to be present long after the stroke has settled (26). Depressive symptoms have been shown to have a predictive effect on return-to-work after stroke (27–29). Possible explanations for the increased prevalence of depression post-stroke include: depression being a risk factor for stroke, depression and stroke having common risk factors, depression being a psychological reaction to stroke or outcomes of stroke (e.g., cognitive impairment, physical disability), and stroke having a direct pathophysiological effect on the brain that leads to changes in imbalances (26).

Post-stroke fatigue occurs in around half of stroke patients, which can persist for over a year after the stroke (30). It is found to be worsened by stress and physical exercise and alleviated by rest (30). Risk factors for post-stroke fatigue include age, being female, being single, cognitive impairment, disability, posterior stroke, inactivity, overweight, alcohol, sleep apnea, and psychiatric issues (e.g., depression, anxiety) (30). A qualitative study with stroke survivors found that fatigue had a devastating influence on their ability to return-to-work (31).

Of the 3 studies which included mood and fatigue management in their intervention programmes (19, 21, 22), 2 studies found positive effects in their outcome measures (21, 22). However, they were both underpowered with small sample sizes. There were several similarities observed between the return-to-work rehabilitation programmes of Ntsiea et al. (22) and Radford et al. (21): (a) the programme was tailored to the individual’s needs and work demands, (b) focus on workplace preparation and skills training, (c) work site visits were conducted and employers were involved in discussion of the return-to-work plan, and (d) provision of fatigue management and psychological support. The remaining studies did not find any positive effects in their outcome measures. Although psychological support was not included in their intervention programme, Cain et al. (18) found less depressive traits to be statistically predictive of return-to-work post-stroke. These findings suggest that mood and fatigue management may potentially be one of the elements of a rehabilitation programme that makes it successful in promoting return-to-work after stroke, however larger scale studies are still needed to support this finding.

In this review, mood and/or fatigue measures were found to be included in 3 studies (18, 19, 21). It is important to measure levels of mood symptoms and fatigue pre- and post-intervention after stroke for several reasons: (a) to screen for mood and fatigue symptoms that may hinder engagement in the intervention, (b) examine if mood and fatigue symptoms predict return-to-work outcomes, and (c) examine if mood and fatigue symptoms improved from the intervention. Studies should also assess for physical recovery during the intervention process, as this is likely to influence mood and /or fatigue outcomes.

A systematic review of psychometric properties and clinical utility of mood screening tools for stroke survivors examined 27 screening tools to identify the most suitable for clinical practise (32). The review identified the observer-rated Stroke Aphasic Depression Questionnaire – Hospital version as having met both psychometric and clinical utility criteria for screening of post-stroke depression. Self-rating scales identified were the Patient Health Questionnaire-9 and Geriatric Depression Scale 15-item to screen for depression, and the Hospital Anxiety and Depression Scale to identify anxiety (32). It is prudent to include more than a single mood measure to screen for both depression and anxiety.

With regards to assessing fatigue, Mead et al. (19) recommended the Fatigue Assessment Scale for use in clinical research. The Fatigue Assessment Scale is a short 10-item self-report scale evaluating symptoms of chronic fatigue, with a high internal consistency of 0.90 (33). It has been used in many diseases including stroke and is the only fatigue measure that has a cut-off score for stroke patients (≥24 indicating post-stroke fatigue) (34).

While return-to-work has been established to be an important rehabilitation goal after stroke with significant benefits, the limited RCTs yielded from this systematic search highlights a dearth of high quality research investigating return-to-work intervention post-stroke. It is encouraging to see that RCTs are gradually emerging in this research field, as seen in this review where 3 out of the 4 studies were published recently. Still, more research is needed to understand the effect of mood and fatigue on return-to-work after stroke, and furthermore to guide the necessary components of a stroke rehabilitation programme for return-to-work.

### 4.1. Limitations

The limitations in this review included the exclusion of non-English studies and small number of participants in 4 of the included studies. Future RCTs involving larger sample sizes are required. Two studies were also assessed to raise some concerns in the risk of bias assessment (20, 21), with Radford et al. (21) feasibility randomised controlled trial which was more descriptive in nature with limited statistical testing. It was also generally difficult to compare the rehabilitation programmes and outcomes between the studies given substantial heterogeneity between the studies in terms of study designs, definition of work, length of follow-up period and outcome measures.

### 4.2. Conclusion

Overall, the findings of this systematic review demonstrated that mood and fatigue are poorly addressed in rehabilitation programmes aimed at improving return-to-work after stroke, despite being a significant predictor of return-to-work. There is limited and inconsistent use of mood and fatigue screening tools. The findings were generally able to provide guidance and recommendations in the development of a stroke rehabilitation programme for return-to-work, including being customised to individual needs, involving work site visits and employers, and use of screening tools. Given the prevalence of mood dysfunction and fatigue post-stroke, it is imperative to include components addressing and measuring psychological support and fatigue management in all post-stroke rehabilitation programmes for improving return-to-work outcomes, to ensure that they are comprehensive and holistic.

## Data availability statement

The original contributions presented in the study are included in the article/Supplementary material, further inquiries can be directed to the corresponding author.

## Author contributions

NC designed the study and drafted the manuscript, with the help of YD. NC and ZK screened and assessed the included studies. ZK and YD reviewed and contributed to the manuscript. All authors contributed to the article and approved the submitted version.

## Funding

YD was supported by the Singapore Medical Research Council (NMRC) Transition Award (NMRC/TA/0060/2017). This publication was supported by SingHealth Group Allied Health.

## Conflict of interest

The authors declare that the research was conducted in the absence of any commercial or financial relationships that could be construed as a potential conflict of interest.

## Publisher’s note

All claims expressed in this article are solely those of the authors and do not necessarily represent those of their affiliated organizations, or those of the publisher, the editors and the reviewers. Any product that may be evaluated in this article, or claim that may be made by its manufacturer, is not guaranteed or endorsed by the publisher.

## Footnotes

^1^ https://www.crd.york.ac.uk/prospero/
