# Supplementary material for: Addressing mood and fatigue in return-to-work programmes after stroke: a systematic review
Source: Front Neurol. 2023 Aug 22;14:1145705. doi: 10.3389/fneur.2023.1145705 (PMC10477595; doi:10.3389/fneur.2023.1145705)
Supplement: Supplementary file 1 [file Data_Sheet_1.PDF]

APPENDIX A: Search Strategy

Scopus – 23 August 2022

Search	Query	Results
1	TITLE-ABS-KEY (("stroke" OR "cerebrovascular accident" OR "cerebral infarction" OR "brain attack" OR "apoplexy") AND ("return to work" OR "employment" OR "job") AND ("rehabilitat*" OR "train*" OR "program*" OR "intervention" OR "protocol") AND ("randomized controlled trial" OR "controlled clinical trial" OR "random*" OR "trial" OR "group" OR "double blind")) AND (LIMIT-TO (PUBSTAGE , "final")) AND (LIMIT-TO (DOCTYPE , "ar")) AND (LIMIT-TO (LANGUAGE , "English")) AND (LIMIT-TO (SRCTYPE , "j"))	323

PubMed – 23 August 2022

Search	Query	Results
1	((Stroke[MeSH Terms]) OR (cerebrovascular accident[MeSH Terms]) OR (cerebral infarction[MeSH Terms]) OR (brain attack[MeSH Terms]) OR (apoplexy[MeSH Terms]) AND ((return to work) OR (employment) OR (job)) AND (((rehabilitat*) OR (train*) OR (program*) OR (intervention) OR (protocol)) AND ((randomized controlled trial) OR (controlled clinical trial)) OR (random*) OR (trial) OR (groups) OR (double blind)))<br>Filters: Full text, English	267

Medline – 23 August 2022

Search	Query	Results
1	exp Stroke/	162655
2	('stroke' or 'cerebrovascular accident' or 'cerebral infarction or 'brain attack' or 'apoplexy').mp.	374609
3	1 or 2	382151
4	exp Return to Work/	3414
5	('return to work' or 'employment' or 'job').mp.	188452
6	4 or 5	188452
7	('rehabilitation' or 'training' or 'programme' or 'intervention' or 'protocol').mp.	1948354
8	Randomized Controlled Trial/	575518
9	('randomized controlled trial' or 'controlled clinical trial' or 'randomized' or 'trial' or 'groups' or 'double blind').mp.	3710674
10	8 or 9	3710674
11	3 and 6 and 7 and 10	124

PsycInfo – 23 August 2022

Search	Query	Results
1	('stroke' or 'cerebrovascular accident' or 'cerebral infarction or 'brain attack' or 'apoplexy').mp. [mp=title, abstract, heading word, table of contents, key concepts, original title, tests & measures, mesh word]	41,006
2	exp Reemployment/	1,743
3	('return to work' or 'employment' or 'job').mp. [mp=title, abstract, heading word, table of contents, key concepts, original title, tests & measures, mesh word]	172,080
4	2 or 3	172,197
5	exp Rehabilitation/ or exp Cognitive Rehabilitation/ or exp Psychosocial Rehabilitation/ or exp Neuropsychological Rehabilitation/	53,268
6	('rehabilitation' or 'training' or 'programme' or 'intervention' or 'protocol').mp. [mp=title, abstract, heading word, table of contents, key concepts, original title, tests & measures, mesh word]	689,531
7	5 or 6	697,211
8	exp Randomized Controlled Trials/ or exp Clinical Trials/	13,309
9	('randomized controlled trial' or 'controlled clinical trial' or 'randomized' or 'trial' or 'groups' or 'double blind').mp. [mp=title, abstract, heading word, table of contents, key concepts, original title, tests & measures, mesh word]	751,169

10	8 or 9	753,605
11	1 and 4 and 7 and 10	44

Embase – 23 August 2022

Search	Query	Results
1	'stroke'/exp OR stroke	634,254
2	'stroke':ab,ti OR 'cerebrovascular accident':ab,ti OR 'cerebral infarction':ab,ti OR 'brain attack':ab,ti OR 'apoplexy':ab,ti	476,008
3	#1 AND #2	459,824
4	'return to work'/exp OR 'return to work'	16,829
5	'return to work':ab,ti OR 'employment':ab,ti OR 'job':ab,ti	170,900
6	#4 AND #5	14,118
7	'rehabilitation'/exp OR rehabilitation	972,013
8	'rehabilitation':ab,ti OR 'training':ab,ti OR 'programme':ab,ti OR 'intervention':ab,ti OR 'protocol':ab,ti	2,430,393
9	#7 AND #8	382,376
10	'randomized controlled trial'/exp OR 'randomized controlled trial'	974,028
11	'randomized controlled trial':ab,ti OR 'controlled clinical trial':ab,ti OR 'randomized':ab,ti OR 'trial':ab,ti OR 'groups':ab,ti OR 'double blind':ab,ti	4,496,332
12	#10 AND #11	741,427
13	#3 AND #6 AND #9 AND #12	18

Cochrane Central Register of Controlled Trials – 30 August 2022

Search	Query	Results
1	MeSH descriptor: [Stroke] explode all trees	11756
2	("cerebrovascular accident") OR (cerebral infarction) OR (brain attack) OR (apoplexy)	21685
3	MeSH descriptor: Return to Work] explode all trees	269
4	(return to work) OR (employment) OR (" Job")	14679
5	("rehabilitation") OR ("training") OR ("programme") OR ("intervention") OR ("protocol")	871605
6	MeSH descriptor: [Randomized Controlled Triall explode all trees	118
7	("randomized controlled trial") OR ("controlled clinical trial") OR ("randomized") OR ("trial") OR ("groups") OR ("double blind")	1568172
8	#1 OR #2	30183
9	#3 OR #4	14679
10	#6 OR #7	1568172
11	#8 AND #9 AND #10 AND #5	237

Web of Science – 30 August 2022

Search	Query	Results
1	((ALL=(return to work)) OR ALL=(employment)) OR ALL=(job)	591630
2	((((ALL=(stroke)) OR ALL=(cerebrovascular accident)) OR ALL=(cerebral infarction)) OR ALL=(brain attack)) OR ALL=(apoplexy)	747780
3	((((ALL=(rehabilitation)) OR ALL=(training)) OR ALL=(program*)) OR ALL=(intervent*)) OR ALL=(protocol)	11431117
4	(((((ALL=(randomized controlled trial)) OR ALL=(controlled clinical trial)) OR ALL=(random*)) OR ALL=(trial)) OR ALL=(groups)) OR ALL=(double blind)	8579695
5	#1 AND #2 AND #3 AND #4	763
6	#1 AND #2 AND #3 AND #4 and English (Languages)	741
7	#1 AND #2 AND #3 AND #4 and English (Languages) and Article (Document Types)	640
